# Association between DRD2/ANKK1 rs1800497 C > T polymorphism and post-traumatic stress disorder susceptibility: a multivariate meta-analysis

**DOI:** 10.3389/fnins.2023.1102573

**Published:** 2023-05-18

**Authors:** Yu-Ming Niu, Jie Zhang, Hong Tang, Lu-Hua Cao, Ting-Yun Jiang, Yuan-Yuan Hu

**Affiliations:** ^1^Department of Stomatology and Center for Evidence-Based Medicine and Clinical Research, Gongli Hospital of Shanghai Pudong New Area, Shanghai, China; ^2^Department of Psychiatry and Joint Laboratory of Psychiatric Genetic Research, The Third People's Hospital of Zhongshan, Zhongshan, Guangdong Province, China; ^3^Department of Psychiatry, Gannan Medical University, Ganzhou, Jiangxi Province, China; ^4^Information Department, Gongli Hospital of Shanghai Pudong New Area, Shanghai, China

**Keywords:** dopamine receptor D2, polymorphism, multivariate analysis, susceptibility, post-traumatic stress disorder

## Abstract

**Background:**

Previous studies have suggested that the DRD2/ANKK1 rs1800497 C > T polymorphism plays a critical role in the risk of post-traumatic stress disorder (PTSD). However, published data are inconsistent or even contradictory. Therefore, we conducted a meta-analysis to explore the underlying correlation between the rs1800497 C > T polymorphism and PTSD risk.

**Materials and methods:**

A total of five online databases were searched, and all related studies were reviewed up to 1 October 2022. Critical information was extracted, and quality assessment was conducted for all included studies. Multivariate meta-analyses were performed for the genetic model choice, and the odds ratios (ORs) and corresponding 95% confidence intervals (CIs) were calculated to examine the statistical power of the genetic models. In addition, heterogeneity, sensitivity, cumulative analysis, and publication bias were analyzed to guarantee statistical power.

**Result:**

Overall, 12 observational studies involving 5,515 subjects were included and analyzed in this meta-analysis. Multivariate analysis indicated that a co-dominant genetic model was most likely the best choice. Pooled results revealed an elevated PTSD risk in mutated homozygote TT carriers in the general population (TT vs. CC: OR = 1.73, 95% CI = 1.14–2.62, *P* = 0.01, *I*^2^ = 58.9%) and other specific subgroups. Moreover, similar results were observed in other genetic models using univariate analysis.

**Conclusion:**

Current evidence suggests that the DRD2/ANKK1 rs1800497 C > T polymorphism may contribute to PTSD susceptibility.

## 1. Introduction

Post-traumatic stress disorder (PTSD) is one of the most important and severe mental disorders occurring in people who have been exposed to a series of traumatic events such as natural disasters, traffic accidents, war, or violent injuries (Javidi and Yadollahie, 2012; Lewis et al., 2019). This disorder shows complex symptoms involving flashbacks, nightmares, and severe anxiety, as well as uncontrollable thoughts about the traumatic event, which can last for months or even years and interfere with daily life and work (Shalev et al., 2017). In the United States, the prevalence of PTSD among adults is 6%, and it is more common among women (9.7%) than among men (3.6%) (Harvard Medical School, 2007). As a serious psychological disorder, PTSD is not only an individual catastrophe but can also lead to serious psychological and financial burdens to the family and society (Hoppen et al., 2021). Although comprehensive etiological research has been conducted on PTSD, the underlying mechanisms of this disorder remain unclear.

Human dopaminergic neurons secrete a variety of hormones that regulate emotional behavior and are correlated with neuropsychiatric disorders (Grandy et al., 1989). Recent evidence had indicated a strong relationship between abnormal dopamine (DA) expression and mental or central nervous system diseases (Frankle et al., 2022; Joshi et al., 2022; Wenceslau et al., 2022). The dysregulation of dopaminergic function and signaling has been found to participate in several psychiatric disorders, such as attention-deficit hyperactivity disorder (ADHD) (Kim et al., 2018), alcohol dependence (Gorwood et al., 2000), schizophrenia (Hussain et al., 2020), and anxiety (Lawford et al., 2006).

Dopamine receptor D2 (DRD2) is a G protein-coupled receptor (GPCR) and is one of the most important intermediate transmitters of the dopaminergic system (Ford, 2014). This receptor is involved in dopamine signaling in presynaptic and postsynaptic neurons and is considered a key gene for PTSD (Yin and Chen, 2020). The current research suggests that DRD2 can activate the self-regulated synaptic pruning mechanism through the mTOR signaling pathway, and the deficiency of synaptic pruning in adolescents results in hyperglutamate function and anxiety-like behavior in adulthood (Yin and Chen, 2020; Zhang and Lin, 2021). Abnormal DRD2 expression and dopamine signaling dysfunction in several mental disorders have become critical therapeutic targets of antipsychotic drugs.

The DRD2 gene is located on human chromosome 11q23 and contains nine exons, several introns, and a short nucleotide repeat (STR) (Eubanks et al., 1992). To date, several single-nucleotide polymorphism (SNP) loci have been shown to be associated with PTSD susceptibility. Among these, rs1800497 C > T is the most common polymorphism locus, which is located 10.5 kb downstream of DRD2 and adjacent to the ankyrin repeat and kinase domain containing one gene (ANKK1) (Yin and Chen, 2020). This polymorphism encompasses a substitution of cytosine to thymine, and the minor T allele (the same as the A1 allele) always combines with the reduced number of dopamine-binding sites in the brain, resulting in a reduced level of dopaminergic activity (Pohjalainen et al., 1998). A recent neuroimaging study showed an association between the rs1800497 polymorphism and hippocampal function and volume, as well as PTSD severity. This was more specifically observed in TC carriers with reduced left CA3 volume and severe PTSD symptomatology (Yuan et al., 2022). In 1991, Comings et al. (1991) conducted the first case–control study on the association between the rs1800497 C > T polymorphism and PTSD susceptibility and reported a negative result in the US population. Since then, numerous studies have been published, but the results have been inconsistent or conflicting. Considering the uncertainty of the current results among published studies, we conducted a meta-analysis to assess the association between the rs1800497 C > T polymorphism and PTSD susceptibility.

## 2. Materials and methods

The review and meta-analysis were performed in accordance with the guidelines of the Preferred Reporting Items for Systematic Reviews and Meta-Analyses (PRISMA) statement (Moher et al., 2009). All collected information was extracted from publications, and no ethical issues were involved.

### 2.1. Literature search

A total of five electronic databases were searched from inception to 1 October 2022 to identify studies focusing on the association between the rs1800497 polymorphism and PTSD susceptibility. The reference lists of the included studies were manually searched to identify additional relevant studies. The retrieval strategy is as follows (e.g., in PubMed):

#1 Dopamine receptor D2;

#2 DRD2;

#3 rs1800497;

#4 Taq1A;

#5 #1 OR #2 OR #3 OR #4;

#6 Polymorphism;

#7 Variant;

#8 Mutation;

#9 #6 OR #7 OR #8;

#10 Post-traumatic stress disorder;

#11 PTSD;

#12 #10 OR #11;

#13 #5 AND #9 AND #12;

### 2.2. Inclusion and exclusion criteria

All retrieved studies were screened with the following criteria for inclusion in this meta-analysis: (1) observational studies on the association between rs1800497 polymorphism and PTSD susceptibility; (2) sufficient genotype data for case and control groups; (3) published studies in English or Chinese; (4) studies with the latest or largest sample size were retained with multiple publications having similar data; and (5) subgroup analyses were conducted only when two or more studies were excluded. The exclusion criteria were as follows: (1) case report and review studies; (2) biological fundamental studies; and (3) studies without sufficient information on genotypes.

### 2.3. Data extraction

Dr. Niu and Zhang reviewed all selected studies and extracted information independently: the surname of the author, year of publication, country and ethnic distribution, control design (population-based control, PB and trauma exposure without PTSD, TEWOP), genotyping method, sample sizes of case and control groups, frequencies of the genotype distribution in both case and control groups, causes of trauma, different crowds, diagnostic criteria, the *p*-value of the Hardy–Weinberg equilibrium (HWE) test in the control group, and NOS scores.

### 2.4. Quality assessment

A total of six departments of representativeness of cases, control design, HWE status in controls, genotyping methods, subject size, and association assessment were independently evaluated by two authors according to the modified Newcastle–Ottawa quality scale (NOS) (Niu et al., 2015). The scores ranged from 0 to 11, and studies with more than 8 points were considered to have high quality (Supplementary Table 1).

### 2.5. Statistical analysis

Crude odds ratios (ORs) and 95% confidence intervals (CIs) were calculated to determine the association between the rs1800497 polymorphism and PTSD susceptibility. The two pooled logORs of log (CT vs. CC) and logOR (TT vs. CC) were first calculated; then, the ratio λ was calculated using the following formula: λ = log(AG vs. AA)/log(GG vs. AA). The genetic model was inferred and calculated according to the ratio λ when the value of λ was equal to 0, 0.5, and 1, which corresponded to the recessive, co-dominant, and dominant models, respectively. An over-dominant model was adopted when the ratio λ was <0 or >1. Furthermore, the statistical power of the other genetic models was determined using univariate analysis regarding allele contrast (T vs. C), the co-dominant model (CT vs. CC and TT vs. CC), the dominant model (CT + TT vs. CC), and the recessive model (TT vs. CC + CT). Potential heterogeneity among the collected studies was examined using *I*^2^ tests. A random-effects model was used when *I*^2^ > 50%; otherwise, a fixed-effects model was employed. Moreover, subgroup analyses were conducted based on HWE status, ethnic distribution, control design, and trauma source. Cumulative meta-analysis and sensitivity analysis were conducted to identify the changing tendency and stability of the results over time. Potential publication biases were assessed using Egger's linear regression test and Begg's funnel plots. All statistical analyses were conducted using STATA (version 14.0; Stata Corporation, College Station, TX, USA) and OpenBUGS 3.2 (http://www.openbugs.net/w/Downloads), and a *p*-value of <0.05 was considered to be statistically significant (two-sided).

## 3. Results

### 3.1. Study characteristics

**Figure 1:**
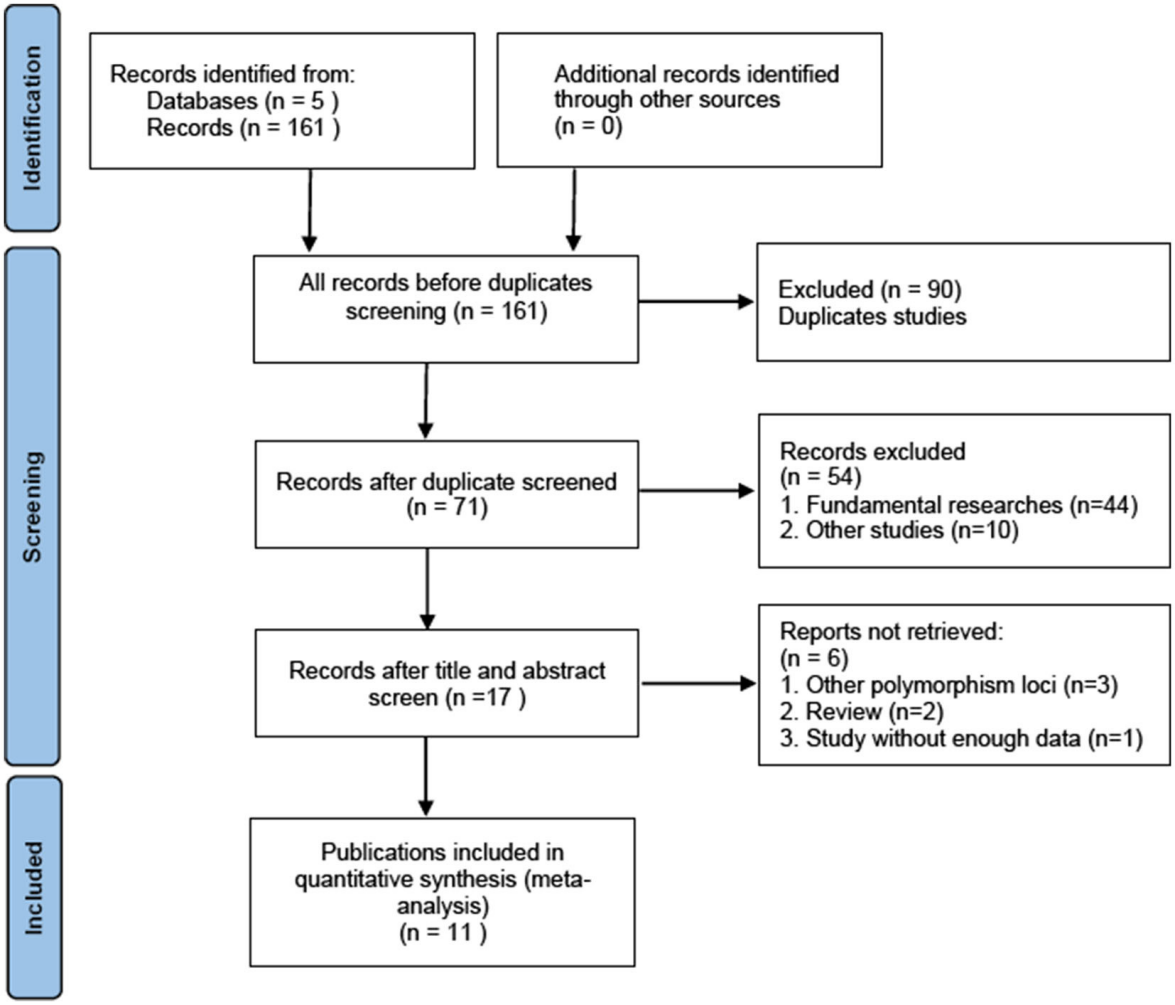
Flow diagram of the study selection process.

The screening process for the included studies is shown in Figure 1. A total of 161 studies were initially identified using the aforementioned search strategy. Thereafter, 90 studies were excluded due to duplicate data. After screening the titles and abstracts, 54 studies were excluded. Then, full-text screening was performed, and six studies were excluded to irrelevant topics. An additional 11 studies were excluded from the review due to lacking complete genotype information. Ultimately, 11 publications with 12 observational studies were included (Comings et al., 1991; Gelernter et al., 1999; Young et al., 2002; Voisey et al., 2009; Li and Wen, 2012; Duan et al., 2015; Tian et al., 2015; Dretsch et al., 2016; Zhang et al., 2018; Hoxha et al., 2019; Xiao et al., 2019).

Among the included studies, six were conducted on Caucasian populations and six on Asian populations. A total of five studies used the polymerase chain reaction-restriction fragment length polymorphism (PCR-RFLP) method, whereas the others used the PCR-ligase detection reaction (PCR-LDR), TaqMan, SnaPShot, and SNPscan methods for polymorphism detection. As control sources, three studies obtained controls from the PB groups, and seven studies used controls from the TEWOP groups. Regarding PTSD causes, war and earthquakes were the most common traumatic factors (Table 1). The *p*-value of HWE of the genotype distributions in the control groups was found to be statistically significant in three studies (Tian et al., 2015; Zhang et al., 2018; Hoxha et al., 2019). In total, four studies had NOS scores of 8 or higher according to the quality assessment criteria. All the information on the included studies is presented in Table 1.

**Table 1:** Characteristics of included studies on DRD2/ANKK1 rs1800497 C > T polymorphism and PTSD risk.

**First author**	**Year**	**Country/region**	**Ethnicity**	**Source of controls**	**Case**	**Control**	**Genotype distribution**						**Genotyping methods**	***P* for HWE**	**Causes of trauma**	**Crowd**	**Diagnostic criteria**	**NOS**
							**Case**			**Control**								
							**CC**	**CT**	**TT**	**CC**	**CT**	**TT**						
Comings	1991	US	Caucasian	Mixed	35	314	19	14	2	237	70	7	DNA-digest	0.50	NA	Civilian	MDRTE	7
Gelernter	1999	US	Caucasian	PB	52	87	37	14	1	56	29	2	PCR-RFLP	0.43	War	Veterans	SCID	7
Young	2002	Australian	Caucasian	HB	91	51	56	34	1	45	5	1	PCR-RFLP	0.10	War	Veterans	DSM-IV	7
Voisey	2009	Australian	Caucasian	PB	127	223	75	47	5	154	61	8	PCR-RFLP	0.53	War	Veterans	DSM-IV	8
Ning	2012	China	Asian	TEWOP	147	200	51	70	26	82	88	30	Snapshot	0.43	NA	Civilian	PCL-C	7
Duan	2015	China	Asian	PB	337	497	128	166	43	174	246	77	PCR-LDR	0.52	Mixed	Civilian	PTSD-RI	11
Tian	2015	China	Asian	TEWOP	64	119	21	26	17	40	70	9	PCR-RFLP	< 0.01	Earthquake	Civilian	PCL-C	6
Dretsch	2015	US	Caucasian	TEWOP	41	189	25	14	2	110	68	10	PCR-RFLP	0.90	War	Veterans	PCL-M	6
Zhang-1	2018	China	Asian	TEWOP	156	978	48	82	26	364	460	154	SNPscan	0.67	Earthquake	Civilian	PCL-5	7
Zhang-2	2018	China	Asian	TEWOP	32	497	14	13	5	227	262	8	SNPscan	< 0.01	Earthquake	Civilian	PCL-5	7
Xiao	2019	China	Asian	TEWOP	287	280	103	132	52	139	108	33	PCR-RFLP	0.10	Earthquake	Civilian	PCL-C	8
Hoxha	2019	Prishtina	Caucasian	TEWOP	364	348	248	107	9	233	112	3	PCR-RFLP	0.01	War	Civilian	M.I.N.I.	8

HWE in control.

PB, population-based control; NA, not available; PCR-LDR, polymerase chain reaction-ligase detection reaction; DSM-IV, diagnostic and statistical manual of mental disorders criteria; PTSD-RI, UCLA posttraumatic stress disorder reaction index criteria; PCL-5, PTSD checklist for DSM-5; PCL-C, PTSD checklist–civilian version; M.I.N.I., mini-international neuropsychiatric interview; PCL-M, PTSD checklist—military version; TEWOP, trauma exposure without PTSD; SCID, structured clinical interviews; PB, population-based control; MDRTE, mental disorders, revised third edition; NA, not available.

### 3.2. Meta-analysis

**Figure 2:**
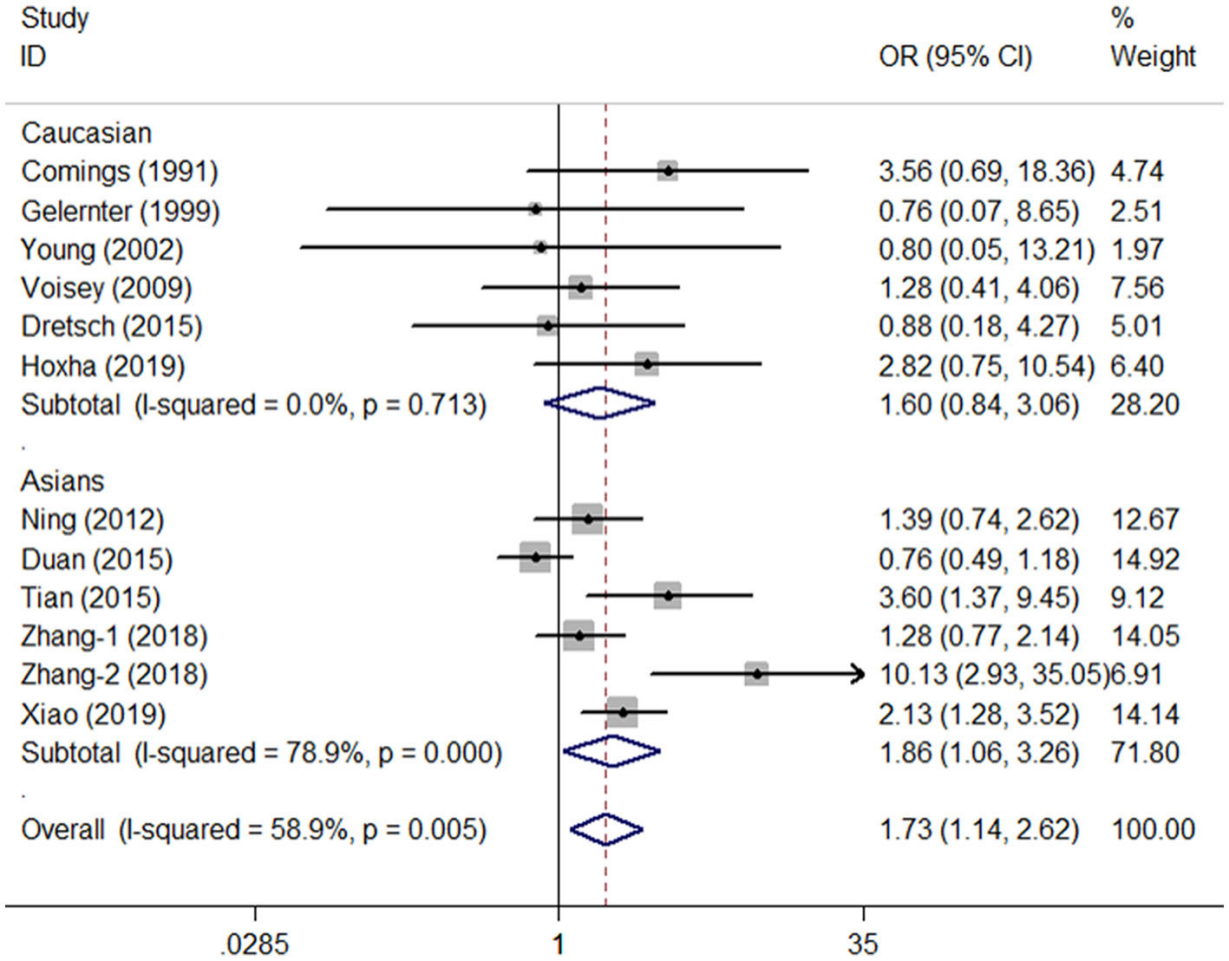
OR and 95% CIs of the associations between DRD2/ANKK1 rs1800497 C > T polymorphism and PTSD susceptibility in the TT vs. CC model.

A total of twelve studies involving 1,733 patients and 3,782 controls were included in our meta-analysis. The estimated λ = 0.512 (95% CI: 0.081–0.954) was close to 0.5, suggesting that a co-dominant genetic model (CT vs. CC and TT vs. CC) of inheritance was more appropriate. Statistical analysis indicated that TT carriers (homozygote model) were associated with elevated PTSD susceptibility in the general population (TT vs. CC: OR = 1.73, 95% CI = 1.14–2.62, *P* = 0.01, *I*^2^ = 58.9%, Figure 2, Table 2). Moreover, many significantly increased risks were found in the co-dominant model in the subgroup analysis based on HWE status (HWE_−yes_: CT vs. CC: OR = 1.38, 95% CI = 1.05–1.82, *P* = 0.02, *I*^2^ = 62.5%; HWE_−no_ TT vs. CC: OR = 4.01, 95% CI = 2.01–7.99, *P* < 0.01, *I*^2^ = 18.9%), Asian groups (TT vs. CC, OR = 1.86, 95% CI = 1.06–2.36, *P* = 0.03, *I*^2^ = 78.9%), control design of TEWOP groups (TT vs. CC, OR = 2.11, 95% CI = 1.33–3.35, *P* < 0.01, *I*^2^ = 55.0%), groups with earthquake experience (TT vs. CC, OR = 2.65, 95% CI = 1.33–5.31, *P* = 0.01, *I*^2^ = 72.7%), and specific crowds of civilians (TT vs. CC, OR = 1.99, 95% CI = 1.20–3.30, *P* = 0.01, *I*^2^ = 72.9%) (Table 2).

**Table 2:** Summary ORs and 95% CI of DRD2/ANKK1 rs1800497 C > T polymorphisms and PTSD risk.

**Locus**	*N* ^*^	**T vs. C**				**CT vs. CC**				**TT vs. CC**				**CT** + **TT vs. CC**				**TT vs. CC** + **CT**			
		**OR**	**95% CI**	***P***	*I*^2^ **(%)**^a^	**OR**	**95% CI**	***P***	*I*^2^ **(%)**^a^	**OR**	**95% CI**	***P***	*I*^2^ **(%)**^a^	**OR**	**95% CI**	***P***	*I*^2^ **(%)**^a^	**OR**	**95% CI**	***P***	*I*^2^ **(%)**^a^
Total	12	1.25	1.06–1.49	0.01	61.9	1.22	0.96–1.54	0.10	61.6	1.73	1.14–2.62	0.01	58.9	1.28	1.02–1.60	0.03	61.2	1.63	1.07–2.48	0.02	64.3
HWE-yes	9	1.26	1.01–1.57	0.04	69.2	1.38	1.05–1.82	0.02	62.5	1.24	0.97–1.57	0.08	29.8	1.39	1.05–1.85	0.02	67.7	1.10	0.89–1.37	0.38	0
HWE-no	3	1.18	0.95–1.46	0.13	30.8	0.85	0.65–1.12	0.26	0	4.01	2.01–7.99	< 0.01	18.9	0.98	0.75–1.27	0.87	0	4.55	2.36–8.77	< 0.01	26.5
**Ethnicity**																					
Caucasian	6	1.32	0.93–1.87	0.12	65.4	1.41	0.87–2.29	0.16	73.7	1.56	0.84–2.93	0.16	0	1.41	0.89–2.22	0.14	72.4	1.46	0.77–2.77	0.25	0
Asian	6	1.23	1.00–1.51	0.05	65.0	1.16	0.98–1.38	0.09	47.9	1.86	1.06–2.36	0.03	78.9	1.22	0.95–1.57	0.11	51.2	1.78	1.02–3.10	0.04	82.0
**Control design**																					
PB	3	1.00	0.72–1.39	0.98	55.8	1.06	0.70–1.60	0.80	56.1	0.81	0.54–1.21	0.30	0	1.03	0.67–1.57	0.90	60.0	0.83	0.57–1.20	0.32	9
TEWOP	7	1.25	1.11–1.41	< 0.01	20.9	1.15	0.97–1.36	0.11	41.0	2.11	1.33–3.35	< 0.01	55.0	1.24	1.05–1.45	0.01	29.0	2.05	1.20–3.52	0.01	71.3
**Causes of trauma**																					
War	5	1.18	0.84–1.67	0.34	58.8	1.27	0.76–2.10	0.36	73.0	1.42	0.73–2.78	0.31	0	1.25	0.79–2.00	0.34	70.2	1.35	0.70–2.63	0.37	0
Earthquake	4	1.37	1.18–1.59	< 0.01	0	1.19	0.83–1.71	0.35	51.2	2.65	1.33–5.31	0.01	72.7	1.43	1.15–1.78	< 0.01	4.7	2.65	1.17–6.01	0.02	83.8
Other	3	1.24	0.81–90	0.34	80.0	1.30	0.79–2.14	0.30	69.2	1.18	0.60–2.29	0.63	59.2	1.33	0.77–2.28	0.31	76.4	0.94	0.68–1.30	0.73	33.6
**Crowds**																					
Veterans	4	1.28	0.78–2.11	0.33	65.1	1.45	0.73–2.91	0.29	73.7	1.04	0.45–2.37	0.93	0	1.41	0.73–2.70	0.30	72.4	0,95	0.42–2.17	0.91	0
Civilians	8	1.25	1.04–1.51	0.02	65.2	1.16	0.91–1.47	0.24	56.4	1.99	1.20–3.30	0.01	72.9	1.24	0.98–1.57	0.07	58.5	1.89	1.14–3.11	0.01	76.6

* Numbers of comparisons.

a Test for heterogeneity.

PB, population-based control; TEWOP, trauma exposure without PTSD.

In addition, some significantly increased PTSD risks were also observed in the general population and subgroup analyses in different genetic models *via* univariate analyses (Table 2).

### 3.3. Cumulative analysis and sensitivity analysis

**Figure 3:**
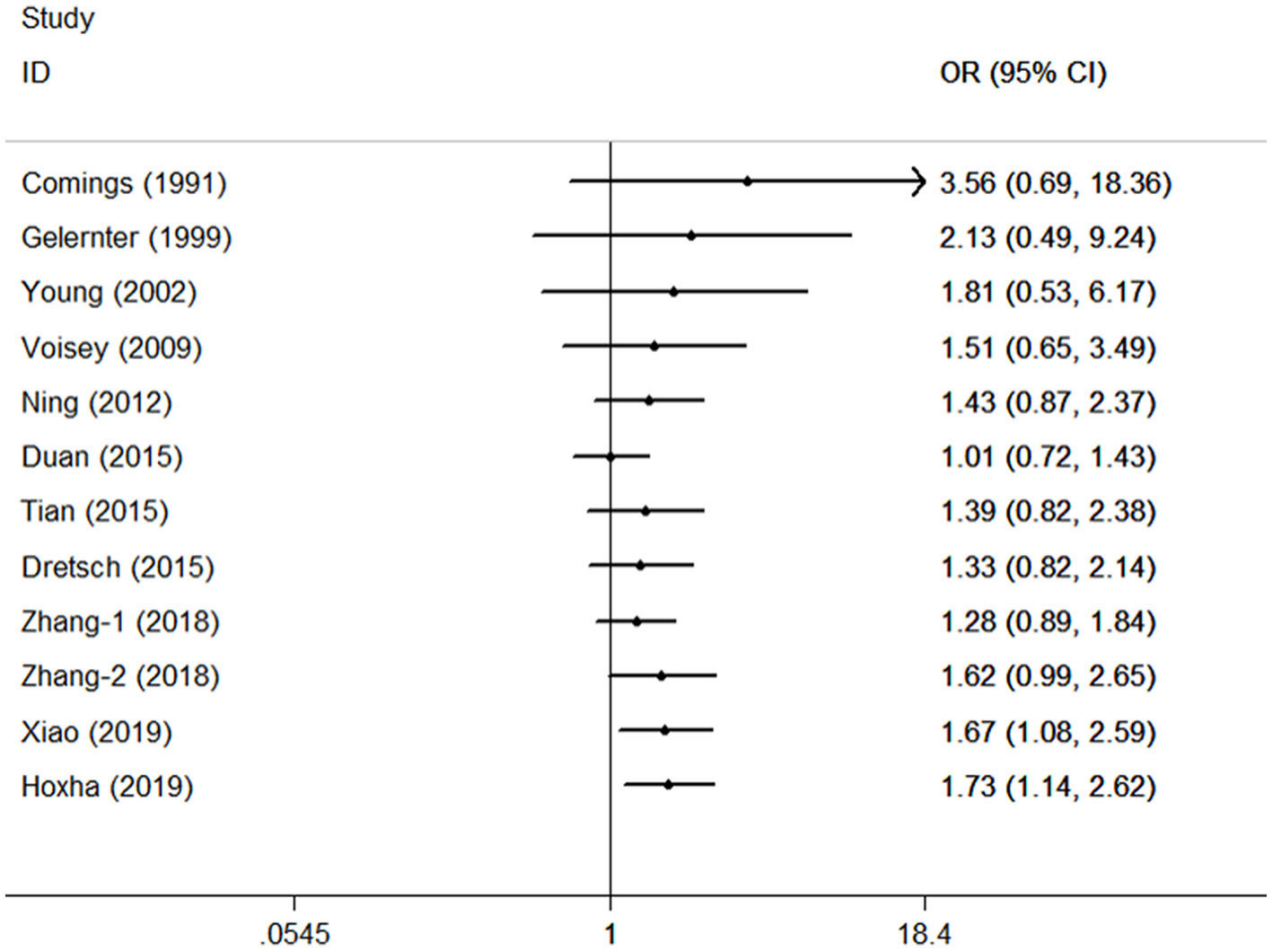
Cumulative meta-analyses according to publication year in the TT vs. CC model of DRD2/ANKK1 rs1800497 C > T polymorphism.

**Figure 4:**
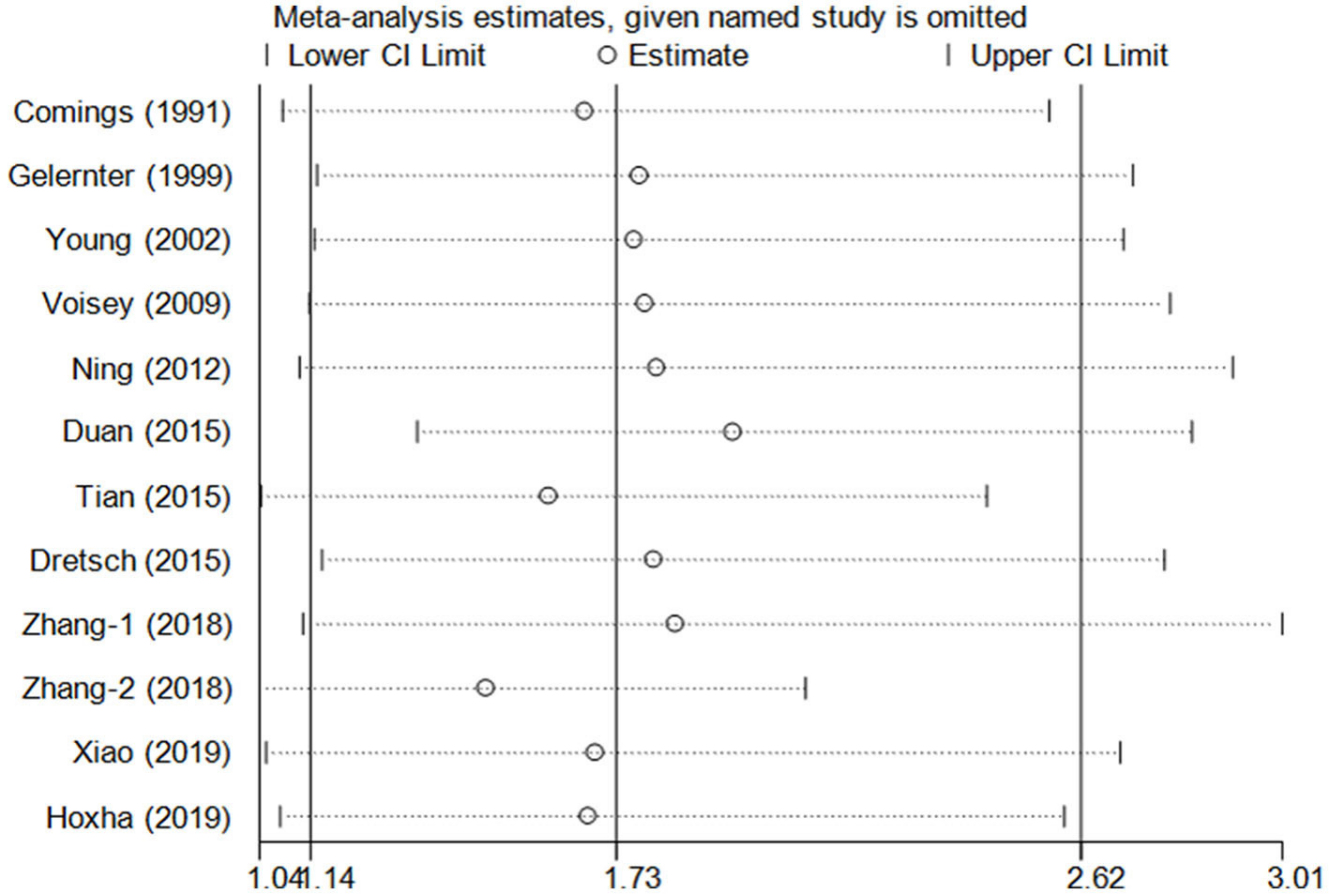
Sensitivity analysis through deleting each study to reflect the influence of the individual dataset on the pooled ORs in the TT vs. CC model of DRD2/ANKK1 rs1800497 C > T polymorphism.

Cumulative analysis showed no fluctuation in the results of recent studies, which showed a progressive increase in PTSD risk (Figure 3 for TT vs. CC model). Sensitivity analyses provided results consistent with the removal of each study according to its publication date (Figure 4 for TT vs. CC model).

### 3.4. Publication bias

**Figure 5:**
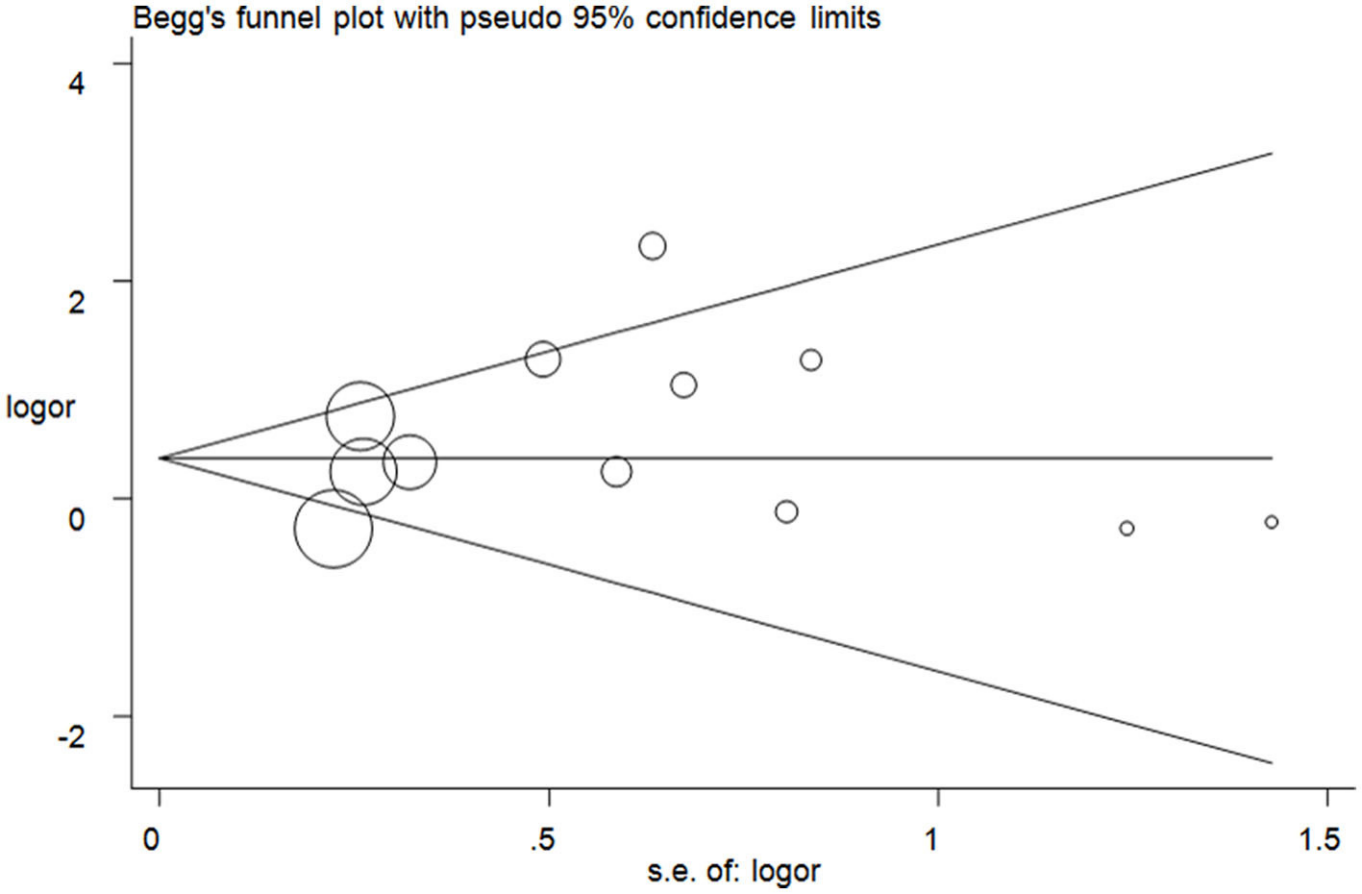
Funnel plot analysis to detect publication bias for the TT vs. CC model of DRD2/ANKK1 rs1800497 C > T polymorphism. Circles represent the weight of the studies.

The publication bias test was assessed, and no significant asymmetry was observed in the funnel plots (Figure 5 for TT vs. CC model). The results were checked with Egger's test (T vs. C, *P* = 0.14; TT vs. CC, *P* = 0.43; CT + TT vs. CC, *P* = 0.26; CT + TT vs. CC, *P* = 0.29; TT vs. CC + CT, *P* = 0.22).

## 4. Discussion

Post-traumatic stress disorder is a common mental disease, and its incidence rate is gradually increasing. It is estimated that 7–8% of the global population suffers from PTSD at some point in their lives (Keane et al., 2006). PTSD always develops after exposure to a highly traumatic stress event. Violent personal assaults, natural or human-caused disasters, accidents, combat, and other forms of violence may be the most important triggers for PTSD. Many people often experience the impact of highly traumatic events, but only a small number eventually experience PTSD. Beyond intrinsic and environmental factors, increasing evidence suggests that genetic factors play a major role in the development and pathogenesis of PTSD. As a member of GPCR, DRD2 can inhibit the activity of adenylate cyclase, regulate the calcium potassium channel activity in neurons, and affect neuronal excitability and function. DRD2 belongs to the family of inhibitory D2-like receptors, which are mainly expressed at the synaptic end of dopaminergic neurons (Zhang and Lin, 2021). Tabano et al. (2022) found that DRD2 methylation levels can be modulated by stress conditions and are positively correlated with PTSD occurrence.

SNPs are the most common type of gene mutation in humans and contribute to individual susceptibility to several diseases through altered gene transcription, expression, and spatial structure. Some studies have suggested that the rs1800497 polymorphism is always accompanied by an abnormal expression of the DRD2 gene in the brain tissue and is associated with several psychiatric disorders, such as anxiety, depression, schizophrenia (Zhou et al., 2022), bipolar disorder (Zhang et al., 2021), and autism spectrum disorder (Liu, 2020). Rs1800497 was originally considered the DRD2 polymorphism locus in most published studies since it could regulate the synthesis of dopamine and reduce D2 receptor expression (Munaf et al., 2009). Thompson et al. (1997) and Jönsson et al. (1999) proved that individuals with the T allele show a decrease of 30–40% in DRD2 density in the striatum compared to wild-type genotype carriers, which is consistent with PTSD patients with dopamine hypoactivity (Lawford et al., 2006). Moreover, Klein et al. (2007) suggested that the replacement of an acidic amino acid (C allele) by a basic amino acid (T allele) was associated with decreased dopamine D2 receptor density/availability in the brain, resulting in decreased sensitivity to traumatic actions. However, the mechanism by which the rs1800497 polymorphism affects or regulates phenotypic expression remains unclear (Thompson et al., 1997). The rs1800497 locus is far from the RD2 gene and cannot affect the expression of the DRD2 gene directly. Therefore, linkage disequilibrium with other functional loci may be a reasonable explanation for the potential mechanism of lower DRD2 density (Pohjalainen et al., 1998). The TaqIA polymorphism has been speculated to be in linkage disequilibrium with mutations in the DRD2 gene or other adjacent genes, such as the rs6276 or rs6277 locus, which is also associated with a decreased expression of DRD2 mRNA (Jiang et al., 2014).

To date, several studies have been conducted to explore the association between the rs1800497 polymorphism and PTSD risk; however, the results remain inconsistent. Gelernter et al. (1999) conducted an early case-control study in 1999 and suggested that the rs1800497 mutation is unlikely to contribute to PTSD risk in US combat veterans. Voisey et al. (2009) and Dretsch et al. (2016) also reported similar results in Australian and US subjects. In contrast, Ning et al. reported an increased PTSD risk in Chinese individuals with mutated genotypes (homozygote contrast: OR = 1.43, 95% CI = 1.17–3.21; heterozygote contrast: OR = 1.77, 95% CI = 1.52–2.10). Tian et al. (2015) suggested that PTSD patients with earthquake experience in the Wenchuan earthquake in China presented a significantly higher TT homozygote rate than controls. Xiao et al. (2019) also reported increased PTSD risk in individuals with the TT genotype (OR = 2.39, 95% CI = 1.39–4.12). The inconsistent results may result from the following: (1) different PSTD causes; (2) different ethnicities and origin countries; (3) deviations of HWE; (4) different genotype assessment methods; and (5) quality differences in the included studies.

Therefore, how can we obtain a more precise result in the current situation? Meta-analysis may be the best choice to resolve the current inconsistencies among studies with larger pooled samples. In 2016, Li et al. (2016) conducted the first meta-analysis on this subject and suggested a significant association between the rs1800497 polymorphism and PTSD susceptibility. The pooled results were derived from six case-control studies involving only 597 patients with PTSD and 1,155 controls, without any subgroup analysis or other quality assessment. To date, more evidence has emerged, and therefore, we conduct this meta-analysis to more precisely assess the association between the rs1800497 polymorphism and PTSD susceptibility based on more eligible data.

In our meta-analysis, 12 observational studies (from 11 publications) involving 5,515 subjects were included. Scientific statistical methods with multiple analyses were employed, and the co-dominant genetic model was suggested as the most appropriate choice to examine the association between the rs1800497 polymorphism and PTSD susceptibility. Overall, the pooled results indicated a significant association between the rs1800497 polymorphism and PTSD susceptibility in the general population and several specific subgroups, such as control design, causes of trauma, and crowds. For TEWOP controls, our results indicate that the mutation of the rs1800497 polymorphism might be a more dangerous triggering factor when PTSD patients and TEWOP controls face the same traumatic exposures. Considering the causes of trauma, the current results indicated that the earthquake was more strongly correlated with PTSD risk than other factors. This may be because the occurrence of earthquakes is sudden and often unpredictable. In addition, earthquakes are more destructive in depth and breadth, and this damage always involves a wider range of people, and the personnel composition is more complex. For different crowds, the stratified analysis demonstrated that civilians were more vulnerable in the face of traumatic events. This also means that the ability of civilians to tolerate and resist high-intensity traumatic events is weaker than that of military personnel who have systematic training. Moreover, in co-dominant genetic models (heterozygote model and mutant homozygote model), the pooled results revealed a statistically significant difference in the general population and subgroups, which also indicated that the rs1800497 TT genotype could increase PTSD risk with a gene dosage effect compared with the CC genotype and the CT genotype. In terms of ethnic differences, six studies focused on Caucasian and Asian descendants, and the results showed that the T allele may be an independent trigger for PTSD risk in Asians but not in Caucasians. Even so, these results should be interpreted with caution because of the small sample size and obvious heterogeneity in Asian populations.

Inevitably, there were some limitations that should be addressed in our research. First, only one polymorphism locus was analyzed in this meta-analysis, and the interactions with other SNPs and the gene–gene or gene–environment synergistic effects could not be assessed due to the lack of original information. Second, moderate heterogeneity was observed in several genetic models, which might have caused bias in the current results. This heterogeneity was partially alleviated with subsequent stratified analysis, and no significant contributing factor was found in meta-regression analyses. Third, the current studies were mostly conducted with Caucasian and Asian descendants, and whether the results could be extended to the general population remains to be demonstrated. Nevertheless, some advantages of our meta-analysis should be highlighted: (1) Multivariate meta-analyses were applied in this study as a scientific statistical method to select a more appropriate genetic model, (2) complete search strategies and rigorous criteria of inclusion and quality assessment were employed, and (3) cumulative analyses and sensitivity analyses were conducted to identify potential interfering factors and to guarantee the credibility of our results.

## 5. Conclusion

In summary, this meta-analysis suggests that the DRD2/ANKK1 rs1800497 polymorphism may increase the risk of PTSD susceptibility. Due to the insufficient quantity and quality of published research, global, high-quality studies with larger sample sizes should be conducted to further validate the current results.

## Data availability statement

The datasets presented in this study can be found in online repositories. The names of the repository/repositories and accession number(s) can be found below: The datasets generated during and/or analyzed during this study are available from the corresponding author on reasonable request.

## Author contributions

Y-MN, JZ, and Y-YH conceived the study. Y-MN, JZ, and HT searched the databases and extracted the data. Y-MN and L-HC analyzed the data. Y-MN, JZ, T-YJ, and Y-YH wrote the draft of the article. T-YJ and Y-YH reviewed the manuscript. All the authors approved the final manuscript.
