# Supplementary material for: Association between DRD2/ANKK1 rs1800497 C > T polymorphism and post-traumatic stress disorder susceptibility: a multivariate meta-analysis
Source: Front Neurosci. 2023 May 18;17:1102573. doi: 10.3389/fnins.2023.1102573 (PMC10232825; doi:10.3389/fnins.2023.1102573)
Supplement: Supplementary file 1 [file Table_1.DOCX]

Supplement Table 1. Scale for quality evaluation.

| Criteria |  | Score |
| --- | --- | --- |
| **Representativeness of cases**  Consecutive/randomly selected cases with clearly defined sampling frame with time, race, quantity and defined criteria  Not consecutive/randomly selected case or without clearly defined sampling frame with time, race, quantity and defined criteria  Not described |  | 2  1  0 |
| **Source of controls**  Population-based  Hospital-based, TEWOP –based or Mixed-based  Not described |  | 2  1  0 |
| **Hardy-Weinberg equilibrium in controls**  Hardy-Weinberg equilibrium  Hardy-Weinberg disequilibrium  Not available |  | 2  1  0 |
| **Genotyping examination**  Genotyping done under “blinded” condition and repeated again  Genotyping done under “blinded” condition or repeated again  Unblinded done or not mentioned and unrepeated |  | 2  1  0 |
| **Subjects**  Number ≥500  Number <500 |  | 1  0 |
| **Association assessment**  Assess association between genotypes and PTSD risk with appropriate statistics and adjustment for confounders  Assess association between genotypes and PTSD risk with appropriate statistics and without adjustment for confounders  Inappropriate statistics used |  | 2  1  0 |
